# Comparative multiomics analyses reveal the breed effect on the colonic host–microbe interactions in pig

**DOI:** 10.1002/imo2.8

**Published:** 2024-07-04

**Authors:** Liang Huang, Shiqi Luo, Shuqi Liu, Mingliang Jin, Yizhen Wang, Xin Zong

**Affiliations:** ^1^ Key Laboratory of Molecular Animal Nutrition, Ministry of Education, College of Animal Sciences Zhejiang University Hangzhou China; ^2^ Key Laboratory of Animal Nutrition and Feed Science in Eastern China, Ministry of Agriculture, College of Animal Sciences Zhejiang University Hangzhou China

**Keywords:** colon, metagenomic, microbiome, pig breed, transcriptomic

## Abstract

Dysregulation of the gut microbiota often leads to immune‐related disorders, indigestion, or diarrhea. Here, Jiaxing Black (JXB) pig, a local Chinese pig breed known for its great tolerance and digestibility of nutrients, was employed for a metagenomic and transcriptomic integrative analysis to reveal the gut microbiota‐genes and gut microbiota‐pathway interactions. A total of 452 differentially expressed genes, and 174 phyla were found between the JXB and the Duroc × Landrace × Yorkshire (DLY) pigs. Detailed analysis revealed that the differences in colon gene expression signatures between the JXB and DLY are mainly enriched in metabolic and inflammatory responses, with *Lactobacillus* and Lachnospiraceae enriched in DLY and JXB, respectively. Notably, Pacebacteria, Streptophyta, and Aerophobetes were found to participate in the PI3K‐Akt mediated immune response in both pig breeds; however, they only accelerated the metabolism in the intestines of JXB pigs. Moreover, the host could regulate microbe metabolism and immune response by Ig‐like domain‐containing protein and *ITIH2*, *PAEP*, and *TDRD9*, respectively. Taken together, our results revealed both common and breed‐specific regulations of host genes by gut microbiota in two pig breeds.

## Highlights

- The different colon gene expression signatures between the Jiaxing Black (JXB) and Duroc × Landrace × Yorkshire (DLY) are mainly enriched in metabolic and inflammatory responses.
- *Lactobacillus* was dominant in DLY, while Lachnospiraceae was enriched in the JXB. Furthermore, Pacebacteria, Streptophyta, and Aerophobetes were identified as key bacteria associated with the PI3K‐Akt mediated immune response.
- Ig‐like domain‐containing protein was involved in regulating the metabolism of intestinal microbiota, while *ITIH2*, *PAEP*, and *TDRD9* were involved in the NLR‐mediated immune response of intestinal microbiota.

## Graphical abstract

The colon contents of the Jiaxing Black (JXB) and the Duroc × Landrace × Yorkshire (DLY) pigs were collected for transcriptomic and metagenomic analyses. By using Weighted Gene Co‐expression Network Analysis and sparse Canonical correlation analysis, we identified the potential interactions between host genes and gut microbes, indicating that host genes involved in the PI3K‐Akt signaling pathway and metabolic pathways were regulated by gut microbes.

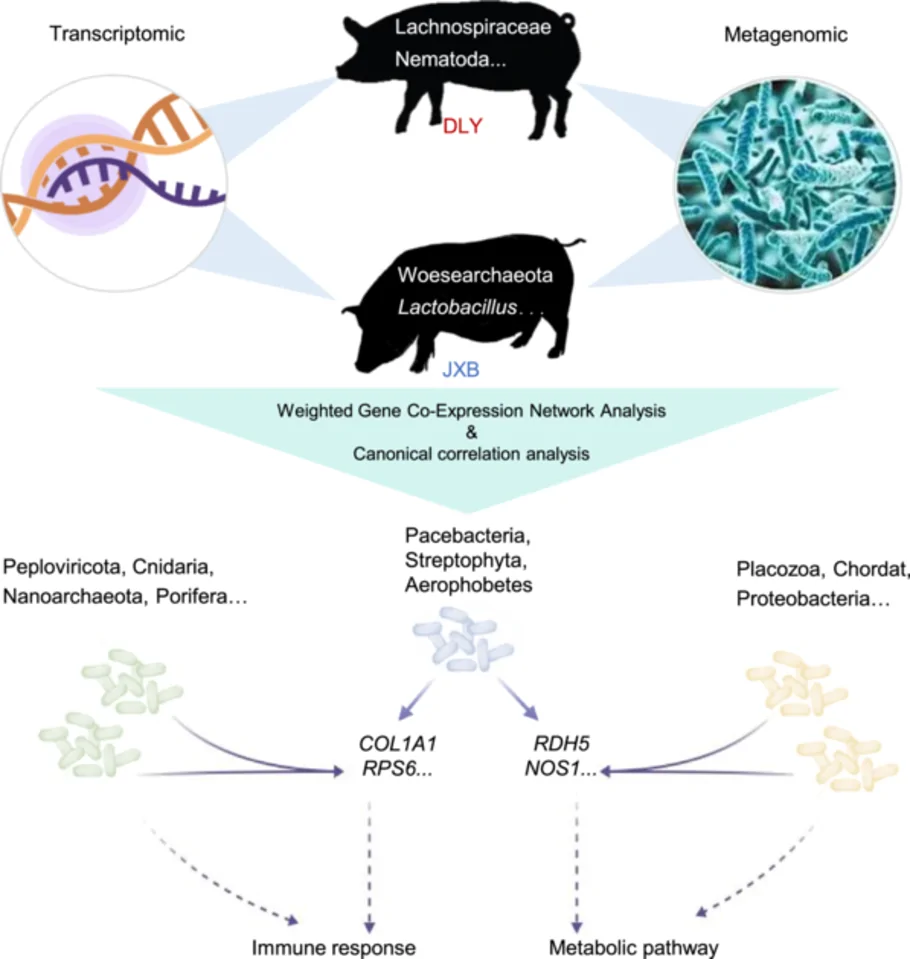

## 1 INTRODUCTION

The gut microbiota coevolves within the host, yet it varies greatly in composition and functions between individuals [1, 2] and critically regulates host metabolism, immune responses, and other key physiological pathways [3, 4]. Gut microbiota homeostasis is closely associated with host diseases [5, 6, 7, 8, 9, 10], particularly metabolic diseases. Dysregulation of the gut microbiota usually causes diseases such as inflammatory bowel disease (IBD) and colitis [11, 12], which are important for personalized medicine treatment.

The structure of the gut microbiome can be modulated by external stimuli, such as diet, antibiotic therapy, and maternal microbiota [2, 13, 14]. Thus, researchers have proposed the hypothesis that the genotype of the host can influence the composition of its microbiota, which in turn affects the host's phenotype as feedback [15, 16, 17, 18, 19, 20]. Given the evidence of such crosstalk, it is critical to detect their interplay to unravel their role in the pathogenesis of human intestinal diseases or animal production. Duroc × Landrace × Yorkshire (DLY) crossbred pigs are commercial pig breeds that are globally selected through advanced breeding to achieve fast growth without compromising disease resistance [21]. Similarly, the Jiaxing Black (JXB) pig is a local pig breed in the Taihu region of China, characterized by its early sexual maturity, high reproductive capacity, coarse feed tolerance, and disease resistance, but with low growth, leanness, and dressing out percentages [22, 23]. These different traits provide an ideal animal model for studying the breed‐mediated microbiota–host interactions. Considering that the colon luminal fluid is characterized by a much longer transit time and a more abundant bacterial population [24, 25, 26, 27], we selected this section of the gut for our study.

To investigate the contribution of colonic bacteria to the pig host, we focused on the interactions between the colonic microbiota and host genes in JXB and DLY pigs. We collected the colon tissue and colonic contents of five individual 190‐day‐old DLY and JXB pigs for RNA‐seq and metagenomic sequencing. As a result, we identified interactions between gut microbes and host genes that are associated with host metabolism and the immune response, providing insight into the breed effects on the host–microbiome interactions.

## 2 RESULTS

### Microbial composition of the colonic microbiota in two distinct breeds

**Figure 1:**
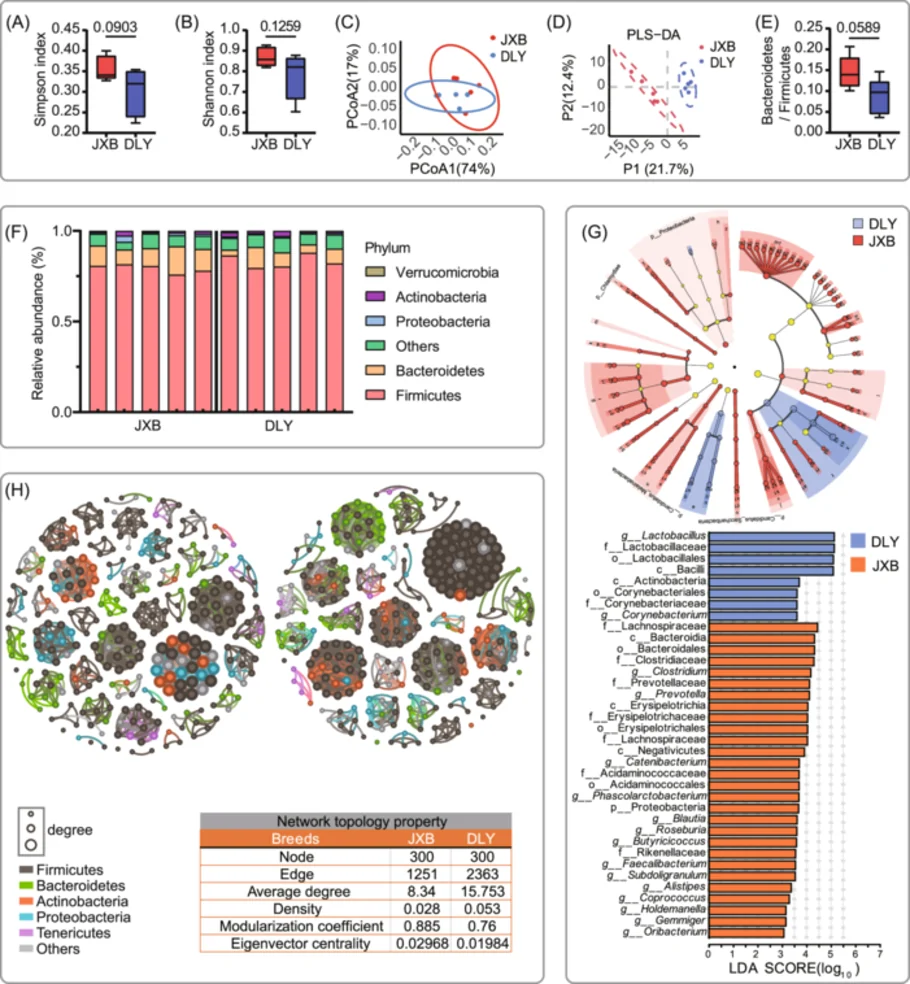
Composition of gut bacteria in the Jiaxing Black (JXB) and Duroc × Landrace × Yorkshire (DLY) pigs (*N* = 5 for each group). (A) The differences in alpha diversity between the two pig breeds are shown by the Shannon and (B) Simpson indices. (C) Principal coordinate analysis (PCoA) and (D) partial least squares discriminant analysis (PLS‐DA) show the differences between the two groups. (E) The ratio of Bacteroidetes to Firmicutes in two groups. The data are presented as the means ± standard error of the mean (SEMs). (F) The relative abundance of the gut flora at the phylum level by stacked plot. (G) The differences in community composition between two groups by Linear discriminant analysis effect size (LEfSe). Nodes with different colors in the figure represent significant effects on the differences between groups, and the yellow nodes indicate microbial taxa without significant changes. The column displays biomarkers at LDA > 3. (H) Visual network and topology statistics of microbial co‐occurrence in the two groups. LDA, linear discriminant analysis.

To detect the different composition of gut microbes, statistical analysis was performed on the metagenomic data of two pig breeds (DLY and JXB), and 208 taxa were detected at the phylum level (Table S1). The Shannon and Simpson indices showed no significant differences in gut microbiota diversity or abundance between these two pig breeds (Figure 1A,B). Principal coordinates analysis (PCoA) also revealed similar beta diversity (Figure 1C). However, partial least squares‐discriminant analysis (PLS‐DA) indicated obvious differences between these two groups (Figure 1D). The relative abundance ratio of Bacteroidetes to Firmicutes was significantly higher in JXB (Figure 1E,F). Using Linear discriminant analysis effect size (LEfSe) to identify the predominant microbial taxa, we showed that *Lactobacillus* was predominant in the DLY, while Lachnospiraceae was enriched in the JXB (Figure 1G and Table S2).

To gain further insights into the colonic microbiota structure of these two breeds, we conducted microbial co‐occurrence network analysis. The correlation among colonic microbes in the DLY gut was more extensive at the genus level than that in the JXB gut (Figure 1H). Notably, the gut microbiota of JXB exhibited a greater modularization coefficient (0.885 vs*.* 0.76) and eigenvector centrality (0.02968 vs*.* 0.01984), suggesting a lower count but a closer interrelationship between them.

A branch of the microbiome, the resistome, consists of antibiotic resistance genes (ARGs) that threaten human health and life. We then investigated the differences in the gut resistome between these two breeds. We identified 304 ARGs in both breeds, and there was no significant difference in the alpha or beta diversity between them (Figure S1A–C). When comparing the relative abundances of the top 10 antibiotic ARGs, macrolide and cephalosporin antibiotic genes were significantly upregulated in the JXB gut, while streptomycin and streptogramin‐a were downregulated (Figure S1D). To reveal the composition structure of ARGs in both breeds, the co‐occurrence of ARGs was determined and showed that bacitracin played a core role in the ARGs of both breeds (Figure S1E,F). The ARGs with the greatest contribution were further determined by a random forest analysis, showing that the top 30 ARGs were displayed in the area under the receiver operating characteristic (ROC) curve of 1 (*p* = 0.0625, the 95% confidence interval [CI] that accuracy better than “no information rate”) (Figure S1G–I).

### Comparison of colon gene expression signatures between DLY and JXB pigs

To detect the differences in the transcriptome profiles, RNA‐seq was carried out. Genes with an expression level (rpm) above 0.001 were counted, and a total of 24,212 genes were detected (Figure S2A and Table S3). Among them, 21,885 genes were found to be present in both pig breeds, while 1104 and 1223 genes were detected specifically in the JXB and DLY colon, respectively. Principal component analysis (PCA) revealed a small distance (*p* = 0.4415 [PC1] and 0.6471 [PC2]) between the two groups (Figure S2B). To identify the differences, differentially expressed genes (DEGs) with a fold‐change > 2 and *p*‐adjust < 0.05 were identified. Overall, 233 genes were upregulated and 219 genes were downregulated in the JXB breed (Figure S1C,D).

**Figure 2:**
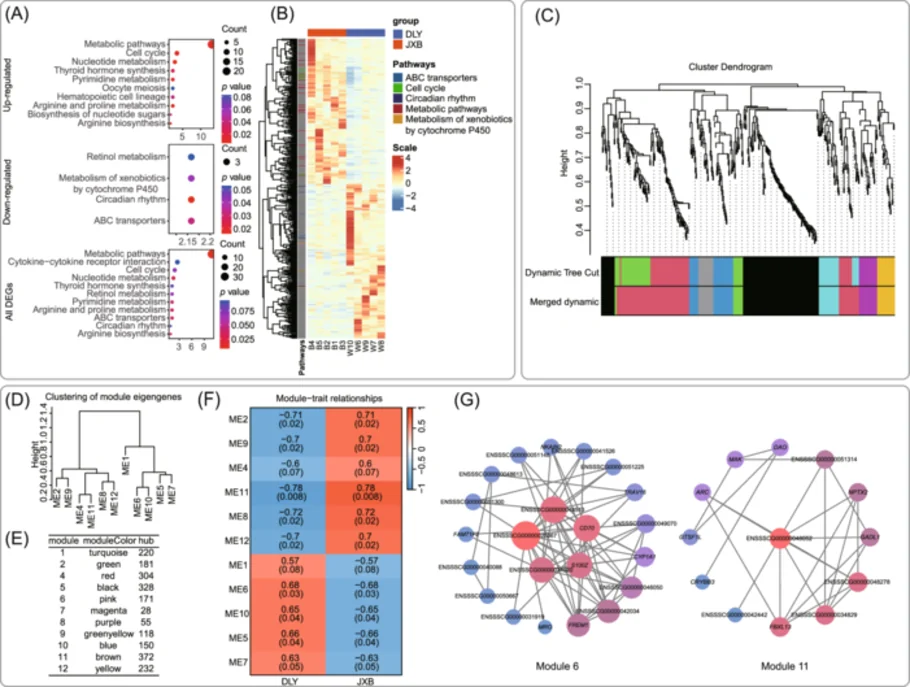
Transcriptional changes of gene sets in the Jiaxing Black (JXB) and Duroc × Landrace × Yorkshire (DLY) pigs (*N* = 5 for each group). (A) Bubble plot shows enriched pathways of differentially expressed genes (DEGs). (B) Heatmap displayed expression of DEGs; pathways they belong to are also marked. (C) The cluster dendrogram. (D) Gene count of each module. (E) Cladogram of modules. (F) Correlations between species and modules by weighted gene co‐expression network analysis (WGCNA). Different colors represent different modules that consist of genes with similar expression modes; *p*‐value was displayed below the correlation coefficient. (G) Network showed the key driver of modules, gradient color, and size of nodes represent degree (edge count of genes); red indicates high degree and blue indicates low degree.

We further performed an enrichment analysis using the Kyoto Encyclopedia of Genes and Genomes (KEGG) to determine the differences in gene functions. The results showed that the upregulated genes in the JXB pigs were mainly enriched in Metabolic pathways compared to those in the DLY pigs (Figure 2A,B). Additionally, pathways such as Nucleotide metabolism and Arginine and proline metabolism were also enriched in the JXB pigs. On the other hand, the downregulated genes in the JXB pigs were mainly enriched in pathways such as Circadian rhythm, ABC transporters, and Metabolism of xenobiotics by cytochrome P450. Integration of all DEGs for enrichment analysis revealed a significant enrichment of Metabolic pathways and Cytokine‐cytokine receptor interaction pathways, highlighting the differences in metabolic and inflammatory responses in the colon tissues of these two pig breeds. For uncovering the DEGs and pathways relationship, their protein interaction network of all DEGs was constructed, but no direct connection was observed between these pathways at the protein level (Figure S2E). In summary, these data suggest that the metabolic levels and immune responses of the JXB pigs differed from those of the DLY pigs, and the metabolic level may have increased due to the upregulated expression of genes involved in metabolic pathways.

To identify the genes contributing to the differences between the JXB and DLY colons, we conducted a weighted gene co‐expression network analysis (WGCNA). Ultimately, 452 DEGs were categorized into 12 modules (Figure 2C,D). Modules 2, 4, 8, 9, 11, and 12 clustered and were identified as JXB marker clusters. Conversely, modules 1, 5, 6, 7, and 10 clustered in the DLY colon (Figure 2E,F). Notably, modules 6 and 11 showed the strongest positive correlation with JXB and DLY pigs, respectively. We supposed that genes within these two modules played a crucial role in driving the disparities between the colons of JXB and DLY pigs. Subsequently, key genes were identified in modules 6 and 11, which demonstrated that *IQCF5* (ENSSSCG00000025367) and *RNA‐directed DNA polymerase* (ENSSSCG00000048052) played a crucial role in their colons (Figure 2G).

### Interactions between host pathways and the gut microbiota

Canonical correlation analysis (CCA) has become a common tool in the analysis of microbiome‐host interaction in recent years [17, 28, 29]. To investigate which microbial taxa are responsible for the differential pathways in the gut of both the JXB and DLY pigs, sparse CCA analysis was carried out with the data of all 10 pigs (five in each breed). The upregulated genes (Up), downregulated genes (Down), and genes whose expression did not change (Common) were individually analyzed. Besides, the genes in the Common group were selected from the top 25% of genes with the highest expression and lowest variance, totaling 4674 genes. Subsequently, different sets of microbiota that interacted with these gene groups were obtained (Tables S4–S6).

**Figure 3:**
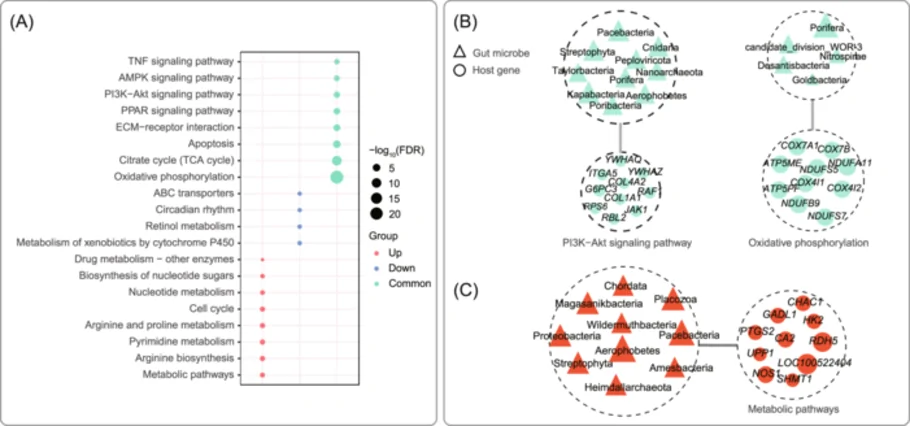
Host pathway–gut microbiota interactions in the Jiaxing Black (JXB) and Duroc × Landrace × Yorkshire (DLY) pigs (*N* = 10 for each gene set). (A) Bubble diagrams show Kyoto Encyclopedia of Genes and Genomes (KEGG) pathways related to enriched genes. Genes were screened by sparse canonical correlation analysis (CCA) (FDR < 0.85) for their interactions with the gut microbiota. (B) Association between the gut flora and host genes in the Common group (genes whose expression did not change in the two breeds). The size of triangles and nodes represents the absolute value of sparse CCA coefficient genes and microbes, respectively. The color of the legend represents each group; red means genes in the Up group (upregulated genes in JXB pigs), and green means genes in the Common group. (C) Association between the gut flora and host genes in the Up group.

According to KEGG enrichment analysis, genes in the Common group were enriched in multiple pathways involved in the immune response and metabolism regulation (Figure 3A). The pathways with the highest numbers of enriched genes and their corresponding interacting microbes were presented (Figure 3B). The genes in the Up group were mostly enriched in metabolic pathways; by interacting with genes (such as *LOC100522404*, *RDH5*, *NOS1*) of the colon, the gut microbes could enhance the metabolism of JXB pigs (Figure 3C). The microbes Pacebacteria, Streptophyta, and Aerophobetes exhibited interactions with both the upregulated genes and commonly expressed genes involved in the PI3K‐Akt signaling pathway and the metabolic pathways, indicating their involvement in gut metabolism and immunity in the JXB pigs.

To investigate the differential interaction between DEGs and the gut microbiota in JXB and DLY pigs, we performed correlation analysis between the 452 DEGs and the gut microbiota in each pig breed and constructed their interaction network, with correlation |cor| > 0.4, *p* < 0.05 regarded as microbe–gene interaction (Tables S7 and S8) (Figure S3A). Based on these, we identified interactions in a breed‐dependent manner (Figure S3A, left and right), as well as the different microbial taxa that interacted with the common genes (Figure S3A, bottom). Among the microbes involved in the interaction, 150 taxa overlapped between these two groups (Figure S3B). We also identified the microbial taxa that exerted regulatory effects on specific DEGs in these two breeds (Figure S3B, left and right) and the DEGs that interacted with a common set of microbes (Figure S3B, bottom). These results illustrated both the similarities and differences in the interactions between differentially abundant bacteria and host genes in two pig breeds.

### Regulation of gut microbiome by host genes

**Figure 4:**
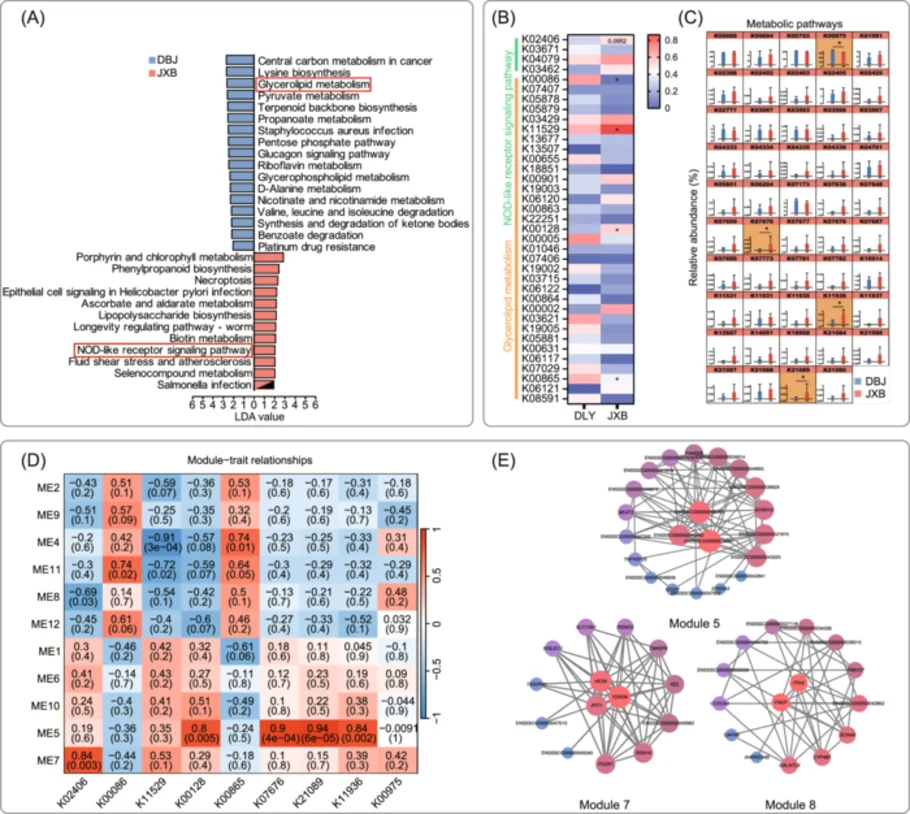
Functional differences of gut microbiota between the Jiaxing Black (JXB) and Duroc × Landrace × Yorkshire (DLY) pigs (*N* = 5 for each group). (A) Linear discriminant analysis effect size (LEfSe) for dominant Kyoto Encyclopedia of Genes and Genomes (KEGG) Ontology in each breed. (B) Heatmap shows different reads counts of KO in NOD‐like receptor signaling pathway and Glycerolipid metabolism; * indicates significant changes at *p* < 0.05 by Mann–Whitney test. (C) Column plot shows reads count of KO involved in metabolic pathways. (D) Correlations between species and modules: two values exist in each cell, representing the correlation coefficient (top) and *p*‐value (bottom). (E) Network shows key drivers of modules: gradient color and size of nodes represent degree (edge count of genes); red indicates high degree, and blue indicates low degree.

To examine the impact of the host genes on the gut microbiome, we analyzed the gut microbiome's functions by comparing reads with the KEGG database. It was observed that the gut microbiome of JXB pigs performed better in the NOD‐like receptor (NLR)‐mediated immune response, as evidenced by the enrichment of reads in the NOD‐like receptor signaling pathway (Figure 4A). Correspondingly, the enriched pathways in the DLY microbiome were primarily associated with metabolism, particularly Glycerolipid metabolism. In addition, pathways such as Valine, leucine, and isoleucine degradation and Synthesis and degradation of ketone bodies were prevalent in DLY pigs, indicating that this breed's improved feed conversion ratio could be attributed to their gut microbiome.

Subsequently, we identified significantly altered subset KEGG orthology (KO) within the NOD‐like receptor signaling pathway and Glycerolipid metabolism and used them to pinpoint related DEG modules (Figure 4B). We specifically selected K02406 from the NOD‐like receptor signaling pathway, as well as K00086, K11529, K00128, and K00865 from Glycerolipid metabolism, and K07676, K21089, K11936, and K11936 from metabolic pathways (Figure 4C) for subsequent correlation analysis, as their reads count were most significantly changed. The data showed that module 5 exhibited a significant correlation with various KOs related to Glycerolipid metabolism and metabolic pathways (Figure 4D). Similarly, modules 7 and 8 were found to be closely correlated with K02406 of the NOD‐like receptor signaling pathway. Consequently, a correlation network was constructed to identify key genes within these modules. Ig‐like domain‐containing protein (ENSSSCG00000034961) was identified as the key host gene in regulating the metabolic function of the DLY gut microbiome, while *ITIH2*, *PAEP*, and *TDRD9* were predominantly involved in regulating NLR‐mediated immune response of the JXB gut microbiome. These results provide a good example of how the host genes modulate the gut microbes.

## 3 DISCUSSION

Research on JXB pigs has focused primarily on meat quality, leading to several omics studies on their muscle [22, 30, 31], yet no research has been conducted on the colon tissue of this breed. Using Yorkshire and Min pigs as a model, a variety of reports have shown that host–microbiota crosstalk promotes a disease‐resistance phenotype [32]. Thus, we asked what type of crosstalk occurs in JXB and how it affects the host phenotype. In this study, we mainly focused on the differences in gut microbe–host interaction related to immunity and metabolism in two breeds, while common interactions in both breeds were also described (as Common group in Figure 3).

The higher abundance of Woesearchaeota and *Lactobacillus* detected in the DLY pig breed is responsible for their stronger digestion and absorption rate [33, 34, 35]. The JXB pig breed has been found to harbor a greater percentage of microorganisms, such as Saccharibacteria, *Nematodes*, and Melainabacteria, especially Lachnospiraceae and Bacteroidia, contributing to its more vibrant community structure [36, 37], stronger antioxidant stress response capabilities [38, 39], and tolerance for coarse feed [40, 41, 42].

The flora that positively interacted with the PI3K‐Akt signaling pathway was more abundant in the JXB colon contents. In addition, Proteobacteria, which have been reported to influence the host serum IgA repertoire and provide structural protection against bacterial sepsis [43, 44], were enriched in the JXB pigs. Placozoa interacts with the Metabolic pathway of the JXB pig gut, which has numerous immune‐related genes and is involved in symbiotic microbiota and resistance to external pathogens [45]. These findings imply a greater intestinal immune response in this breed. Ig‐like domain‐containing protein participates in immune response. Interestingly, we found that it also regulated the metabolism of gut microbes, implying that Ig‐like domain‐containing protein links bacterial metabolism and host immunity. However, further research is required to confirm it, since there is no research report on this topic yet.

The differential interaction patterns between host genes and microbial communities provided initial insights into the underlying mechanisms contributing to the differences in productivity between these two pig breeds. These findings can also serve as a reference for the mechanism understanding of gut microbiota and their potential applications in livestock production. There are still limitations in this research that need to be addressed, and further in‐depth investigations with experimental validations are required to understand the mutual influence and causality between the gut microbiota and host genes.

## 4 CONCLUSION

We constructed a network of pathway‐microbe and gene‐microbe interactions to determine the regulatory effect of gut microbes on the host colonic and host‐to‐gut microbial immune response and metabolism by comparing two pig breeds. The colonic transcripts, *IQCF5*, and *RNA‐directed DNA polymerase*, were the main regulator of global colonic transcription, while Ig‐like domain‐containing protein and *ITIH2*, *PAEP*, and *TDRD9* contribute to the metabolism and immunity in both breeds, respectively. The gut microbes, such as Pacebacteria, Streptophyta, and Aerophobetes, were found to participate in the PI3K‐Akt mediated immune response in both pig breeds; concurrently, they accelerated the metabolism in the intestines of JXB pigs. This research will be helpful for improving livestock productivity as well as intestinal diseases in pigs.

## 5 MATERIALS AND METHODS

### Animals and sample collection

The animal experimental protocols were approved by the Animal Care and Use Committee of Zhejiang University (No. 28812). Five DLY (190 days old) and five JXB pigs (190 days old) were obtained from Zhejiang Qinglian Food Company. These animals were all housed under the same feedlot conditions and were fed the same diet, which consisted of corn (68%), soybean meal (containing crude protein [CP] 46%, 18%), or other feed supplements (total of 14%). Colonic contents from all pigs were collected before 12 h of fasting for slaughter. After slaughter, the colon tissues were collected in tubes and stored at −80°C.

### RNA library construction

Total RNA was isolated from colon tissues with TRIzol reagent (Invitrogen) following the manufacturer's instructions. High‐quality RNA (OD260/280 = 1.8–2.2, OD260/230 ≥ 2.0, RIN ≥ 8.0, 28S:18S ≥ 1.0) was accurately detected using a Bioanalyzer 2100 system (Agilent Technologies) and quantified using an ND‐2000 (NanoDrop Technologies) to construct a sequencing library. RNA purification, reverse transcription, library construction, and sequencing were performed at Shanghai Majorbio Biopharm Biotechnology Co. Ltd. using the TruSeq Stranded mRNA LT Sample Prep Kit (Illumina) as described previously [46]. Briefly, messenger RNA was first isolated by oligo(dT) beads according to the poly(A) selection method and then fragmented with a fragmentation buffer. Second, double‐stranded cDNA was synthesized using a Super Script Double‐Stranded cDNA Synthesis Kit (Invitrogen) with random hexamer primers (Illumina). Then, the synthesized cDNA was subjected to end repair, phosphorylation, and “A” base addition according to Illumina's library construction protocol. Libraries were size selected for cDNA target fragments of 300 bp on 2% low‐range Ultra agarose followed by PCR amplification using Phusion DNA polymerase (NEB) for 15 PCR cycles. After quantification by TBS380, the paired‐end RNA‐seq library was sequenced with an Illumina NovaSeq. 6000 sequencer (2 × 150 bp read length).

### RNA sequencing, alignment, and analysis

The raw paired‐end reads were trimmed and quality‐controlled by fastp (https://github.com/OpenGene/fastp) [47] with default parameters. Then, the clean reads were separately aligned to the reference genome in orientation mode using HISAT2 (http://ccb.jhu.edu/software/hisat2/index.shtml) software [48]. The mapped reads of each sample were assembled by StringTie (https://ccb.jhu.edu/software/stringtie/) via a reference‐based approach [49].

To identify DEGs between two different samples/groups, the expression level of each gene was calculated according to the transcripts per million reads (TPM) method. RSEM (http://deweylab.biostat.wisc.edu/rsem/) was used to quantify gene abundances [50]. Essentially, differential expression analysis was performed using DESeq2 [51], and DEGs with |log_2_ (foldchange)| ≥ 1 and *p*‐adjust ≤ 0.05 (DESeq2/edgeR/Limma)/*p*‐adjust ≤ 0.001 (DEGseq)/Prob > 0.8 (NOIseq) were considered to be significantly DEGs. In addition, functional enrichment analysis with KEGG (http://www.genome.jp/kegg/) was performed to identify which DEGs were significantly enriched in metabolic pathways at *p*‐adjust ≤ 0.05 compared with the whole‐transcriptome background. KEGG pathway analysis was carried out by Goatools (https://github.com/tanghaibao/Goatools) and KOBAS (http://kobas.cbi.pku.edu.cn/home.do) [52]. Interactions were predicted by STRING software (https://string-db.org/) and visualized using Cytoscape v.3.8.2 (Cytoscape Consortium).

### Gut content DNA extraction, library construction, and metagenomic sequencing

Total genomic DNA was extracted from samples using the E.Z.N.A.® Soil DNA Kit (Omega Biotek) according to the manufacturer's instructions. The concentration and purity of the extracted DNA were determined with TBS‐380 and NanoDrop2000, respectively. The quality of the extracted DNA was checked on a 1% agarose gel.

The DNA extract was fragmented to an average size of approximately 400 bp using Covaris M220 (Gene Company Limited) for paired‐end library construction. A paired‐end library was constructed using NEXTflexTM Rapid DNA‐Seq (Bioo Scientific). In brief, the fragmented DNA was combined with End Repair Mix and purified with a QIAquick PCR Purification Kit (QIAGEN). Then, A‐Tailing Mix was added. The purified adenylate 3′‐end DNA was combined with the adapter and ligation mix. Several rounds of PCR amplification with PCR Primer Cocktail and PCR Master Mix were performed to enrich the adapter‐ligated DNA fragments. Finally, PCR amplification was carried out, and PCR products were purified with AMPURE XP beads to obtain the final library.

Paired‐end sequencing was performed on an Illumina NovaSeq/HiSeq Xten platform (Illumina Inc.) at Majorbio Bio‐Pharm Technology Co. Ltd. using NovaSeq Reagent Kits/HiSeq X Reagent Kits according to the manufacturer's instructions (www.illumina.com).

### Metagenomic quality control and preprocessing

The raw reads from metagenome sequencing were used to generate clean reads by removing adapter sequences and trimming and removing low‐quality reads (reads with N bases, a minimum length threshold of 50 bp, and a minimum quality threshold of 20) using fastp [49] (https://github.com/OpenGene/fastp, version 0.20.0) on the free online platform of the Majorbio Cloud Platform (cloud.majorbio.com). The clean reads were mapped to the reference pig genome (Sscrofa 11.1) using BWA (v 0.7.15) to identify and remove the human host‐originating reads [53]. These high‐quality reads were then assembled into contigs using MEGAHIT [54] (parameters: kmer_min = 47, kmer_max = 97, step = 10) (https://github.com/voutcn/megahit, version 1.1.2), which makes use of succinct de Bruijn graphs. Contigs with lengths greater than or equal to 300 bp were selected for final assembly. Finally, 68.2 Gb of high‐quality PE reads were acquired, with an average of 6.82 Gb of clean reads generated for each sample.

### Microbiome gene prediction and gene catalog construction

The reads were assembled into contigs for all samples using the assembly software SOAPdenovo2 (https://github.com/aquaskyline/SOAPdenovo2). Total reads were used to generate 4.9 million contigs without ambiguous bases (minimum length of 300 bp). Open reading frames (ORFs) in contigs were identified using MetaGene [55]. The predicted ORFs with lengths greater than or equal to 100 bp were retrieved and translated into amino acid sequences using the NCBI translation table. The final nonredundant gene set contained 6,808,376 ORFs with an average length of 462 bp.

A nonredundant gene catalog was constructed using CD‐HIT (version 4.6.1) [56], with 90% sequence identity and 90% coverage. After quality control, the reads were mapped to the nonredundant gene catalog with 95% identity using SOAPaligner (version 2.21) [57], and the gene abundance in each sample was evaluated.

### Metagenomic community profiling

To estimate the microbiome species richness of an individual from the taxonomic profiles of the PREDICT one participant, we computed two alpha diversity measures: the number of species found in the microbiome (“observed richness”) and the Shannon entropy estimation. We did not perform rarefaction before alpha diversity calculations because of the low standard deviation in sequencing depths and the verified missing correlation between the metadata of interest and sequencing depth. Microbiome dissimilarity between participants (beta diversity) was computed using the Bray‐Curtis dissimilarity of microbiome taxonomic profiles.

### Metagenomic functional profiling

Functional potential analysis was conducted on quality‐filtered reads using HUMAnN2 (version 0.11.2 and UniRef database release 2014–07) [58], which determines the abundance of genes and pathways in a given Metagenomic community. Microbe co‐occurrence networks and topology properties were drawn and calculated by Gephi. To identify those functional categories that were differentially represented across pig breeds, we employed LEfSe [59], a validated tool that identifies differentially abundant biomarkers such as genes, pathways, or organisms between microbial communities.

### Transcriptomic and metagenomic data integration

The sparse CCA method is clearly described in Priya et al. [17]. In brief, we performed sparse CCA analysis by using the R package “PMA” as a reference. After performing hyperparameter tuning to identify the sparsity penalty parameters and considering too few samples to obtain enough gene and microbe sets, we identified and selected the sparsity penalty parameters λ1 as 0.4, λ2 as 0.4, and *k* as 10 for each sparse CCA analysis. We computed the significance of each pair of components using leave‐one‐out cross‐validation (LOOCV) and identified components whose FDR < 0.85 as effective components. The results are shown in Tables S4–S6. To facilitate visualization, we retained the maximum absolute value coefficient while the same gene appeared in different components and selected the top 10 microbes or genes ranked by the absolute value of the sparse CCA coefficient for host pathway–gut microbe interactions.

We performed Spearman correlation analysis using the corr.test function of the R package “psych” to analyze the interactions between host genes and gut microbes. The associations whose absolute correlation coefficient was >0.4 and *p* < 0.05 between genes and microbes were considered interactions.

### Statistical analysis

Statistical comparisons were performed using nonparametric Kruskal–Wallis tests for the metagenome and two‐tailed *t*‐tests for the transcriptome. Differences were regarded as statistically significant at *p* < 0.05. Hierarchical clustering was conducted using the Bray‐Curtis similarity index via the unweighted pair‐group method with arithmetic averages.

## AUTHOR CONTRIBUTIONS

**Liang Huang**: Writing—original draft; writing—review and editing; visualization; software; formal analysis; conceptualization; validation; methodology. **Shiqi Luo**: Writing—review and editing; resources; formal analysis; writing—original draft; visualization; validation; methodology. **Shuqi Liu**: Writing—review and editing; formal analysis; resources. **Mingliang Jin**: Project administration; supervision. **Yizhen Wang**: Project administration; supervision. **Xin Zong**: Investigation; funding acquisition; conceptualization; writing—review and editing; writing—original draft; project administration; supervision; resources; data curation; methodology; validation.

## CONFLICT OF INTEREST STATEMENT

The authors declare no conflict of interest.

## ETHICS STATEMENT

All animal experiments were approved by the Animal Care and Use Committee and the Animal Experimentation Ethics Committee of Zhejiang University (Hangzhou, China), and the protocols were approved by the Animal Ethics Committee (No. 28812).

## Supporting information


**Figure S1:** Antibiotic resistance genes (ARGs) differ between the two pig breeds (N = 5 for each group).
**Figure S2:** Differentially expressed genes (DEGs) between the two pig breeds (N = 5 for each 594 group).
**Figure S3:** Differential host gene‐gut microbiota interactions between two pig breeds (N = 5 for each gene or microbe set).

**Table S1:** Reads count of microbes at phylum level.
**Table S2:** Linear Discriminant Analysis (LDA) score and p value of dominant microbes by Linear discriminant analysis Effect Size (LEfSe).
**Table S3:** Expression matrix of whole genes quantified by rpm.
**Table S4:** Components of up‐regulated genes (Up) and microbes by sparse canonical correlation analysis (CCA).
**Table S5:** Components of down‐regulated genes (Down) and microbes by sparse canonical correlation analysis (CCA).
**Table S6:** Components of common genes (Common) and microbes by sparse canonical correlation analysis (CCA).
**Table S7:** Correlations between differentially expressed genes (DEGs) and microbes by spearman correlation analysis.
**Table S8:** Correlations that |cor| > 0.4 and p < 0.05 between differentially expressed genes (DEGs) and microbes.

## Data Availability

All the raw data used in this research are available in the NCBI SRA data set (BioProject: PRJNA1005787, at: https://www.ncbi.nlm.nih.gov/bioproject/PRJNA1005787/). The data and scripts used are saved in https://github.com/hillincre/DLY-and-JXB-multi-omics-analysis. Supplementary materials (tables, figures, scripts, graphical abstracts, slides, videos, Chinese translated versions, and updated materials) can be found in the online DOI or iMeta Science http://www.imeta.science/imetaomics/.
