# Supplementary material for: Comparative multiomics analyses reveal the breed effect on the colonic host–microbe interactions in pig
Source: IMetaOmics. 2024 Jul 4;1(1):e8. doi: 10.1002/imo2.8 (PMC12806241; doi:10.1002/imo2.8)
Supplement: Supplementary file 1 — Figure S1: Antibiotic resistance genes (ARGs) differ between the two pig breeds (N = 5 for each group). Figure S2: Differentially expressed genes (DEGs) between the two pig breeds (N = 5 for each 594 group). Figure S3: Differential host gene‐gut microbiota interactions between two pig breeds (N = 5 for each gene or microbe set). [file IMO2-1-e8-s002.docx]

# Supporting information to:

# Comparative multi-omics analyses reveal the breed effect on the colonic host-microbe interactions in pig

**Running title: Host-gut microbe interactions in pig.**

Liang Huang^1,2#^, Shiqi Luo^1,2#^, Shuqi Liu^1,2^, Mingliang Jin^1,2^, Yizhen Wang^1,2^, Xin Zong^1,2*^

^1^Key Laboratory of Molecular Animal Nutrition, Ministry of Education, College of Animal Sciences, Zhejiang University, 310058 Hangzhou, China

^2^Key Laboratory of Animal Nutrition and Feed Science in Eastern China, Ministry of Agriculture, College of Animal Sciences, Zhejiang University, 310058 Hangzhou, China

^#^These authors contributed equally: Liang Huang, Shiqi Luo

*Correspondence: [zongxin@zju.edu.cn](mailto:zongxin@zju.edu.cn) (Xin Zong)

## Supplementary figures


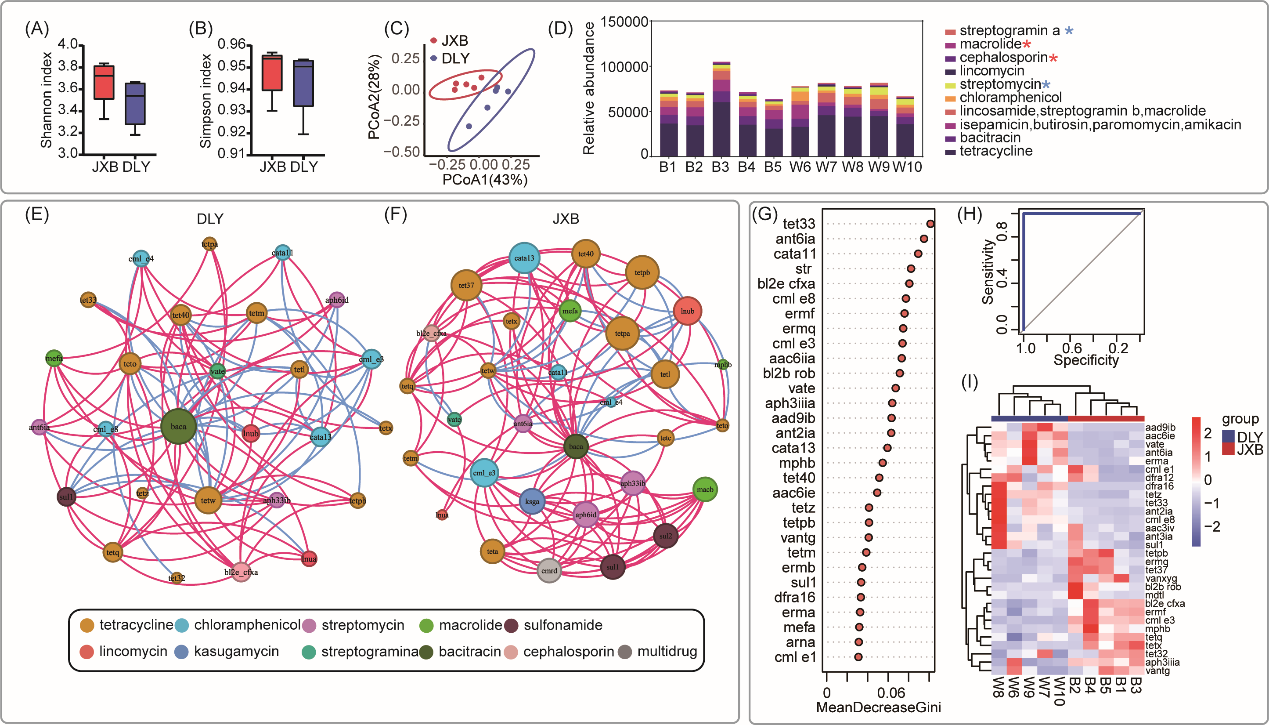


**Figure S1 ARGs (Antibiotic resistance genes) differ between the two pig breeds (N = 5 for each group).** (A) Alpha diversity of ARGs compared by Shannon and (B) Simpson indices. (C) PCoA analysis showed differential beta diversity between the two groups. (D) Stack diagram displaying the top 10 antibiotic types in each pig. * with red indicates up-regulated genes in the JXB group, * with blue indicates down-regulated genes. (E) The co-occurrence networks among ARGs in the DLY and (F) in the JXB. Red and blue edges represent positive and negative correlations, respectively, between nodes. The size of the nodes represents the degree. (G) Random forest filtered the top 30 most important antibiotic types in the two pig breeds and (H) the ROC. (I) abundance heatmap of these genes are shown.


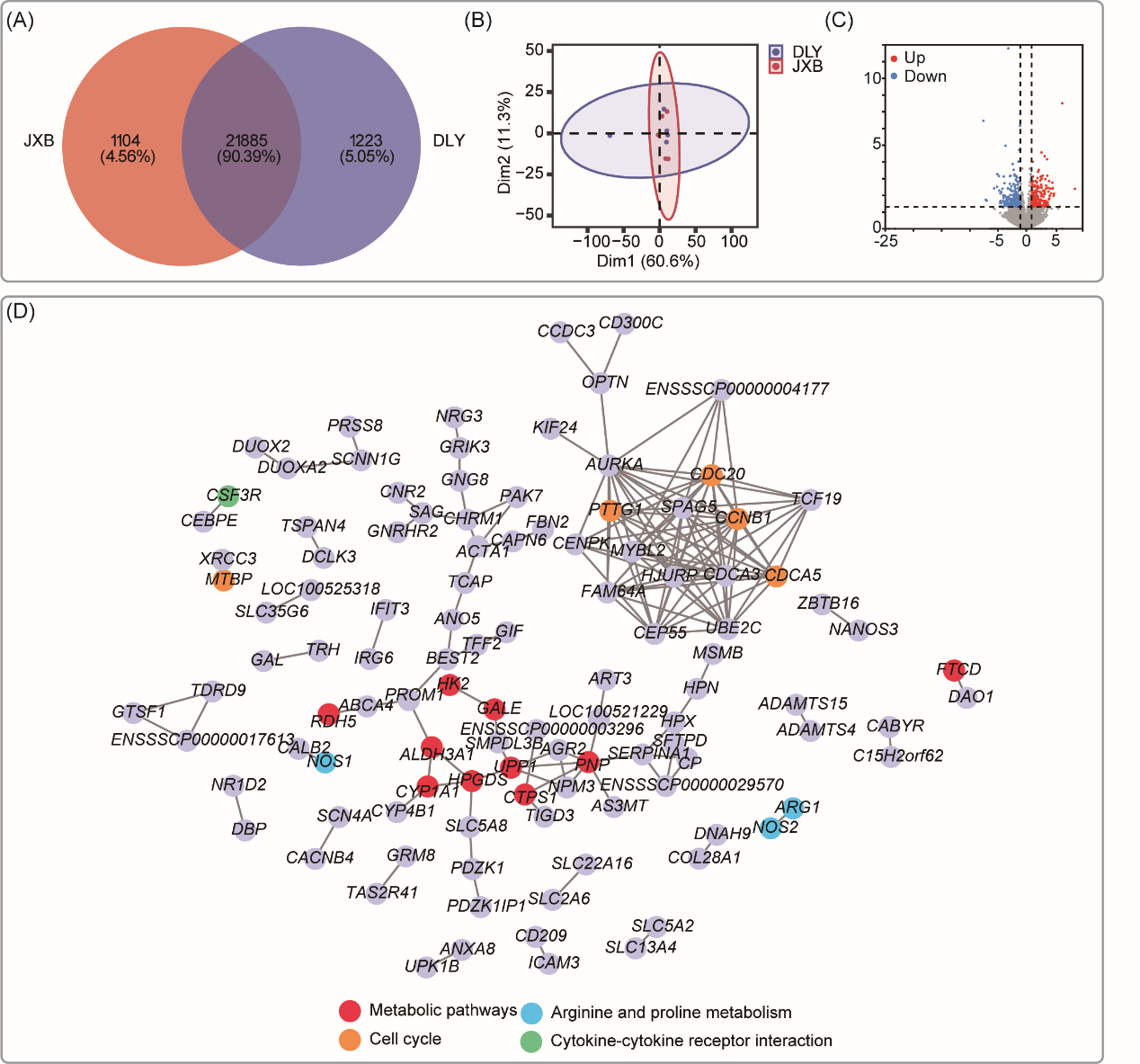


**Figure S2 Differentially expressed genes (DEGs) between the two pig breeds (N = 5 for each group).** (A) Venn diagram show the gene counts of DEGs between two pig breeds. (B) PCA show the distance between two groups. (C) Volcano plot show significantly different DEGs. (D) Network show interactions between proteins, colors represent pathways the proteins belong to.


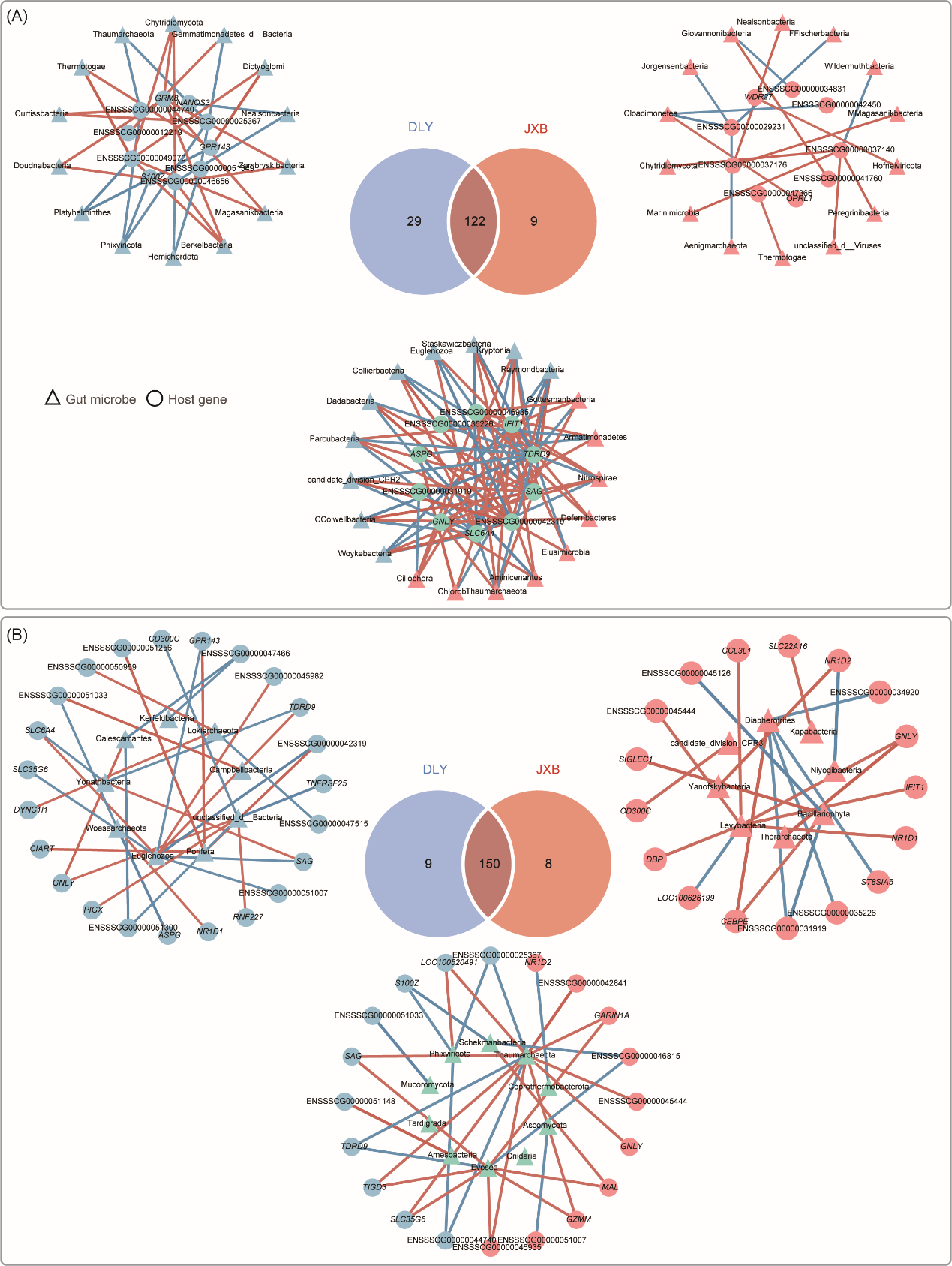


**Figure S3 Differential host gene-gut microbiota interactions between two pig breeds (N = 5 for each gene or microbe set).** (A) Venn diagram show the number of differentially expressed genes that interact with microbes (absolute coefficient > 0.4, *p* < 0.05). Network diagram on the left shows genes that only interact with microbes in DLY and their interacting microbes, and network on the right shows genes that only presented in JXB. Network under the Venn diagram shows genes that are present in both groups and their different interacting microbes. (B) Venn diagram shows counts of differential microbes that interact with microbes (absolute coefficient > 0.4, *p* < 0.05). Network diagram on the left shows microbes that only interact with host genes in DLY and their interacting genes (top 20 ranked by the absolute value of the Spearman correlation coefficient). Network on the right shows microbes that are only present in JXB. Network under the Venn diagram shows microbes that are present in both groups and their different interacting genes.
